# Development of a Novel Machine Learning Model for Automatic Assessment of Quality of Transvaginal Ultrasound Images From Multi‐Annotator Labels

**DOI:** 10.1002/ajum.70026

**Published:** 2025-09-22

**Authors:** Alison Deslandes, Daniel Petashvili, Hu Wang, Gustavo Carnerio, Jodie Avery, George Condous, Mathew Leonardi, M. Louise Hull, Hsiang‐Ting Chen

**Affiliations:** ^1^ Robinson Research Institute University of Adelaide Adelaide Australia; ^2^ School of Computer and Mathematical Sciences University of Adelaide Adelaide Australia; ^3^ Australian Institute of Machine Learning University of Adelaide Adelaide Australia; ^4^ University of Surrey Guildford UK; ^5^ Endometriosis Ultrasound & Advanced Endosurgey Unit, Sydney Medical School Nepean University of Sydney, Nepean Hospital Sydney New South Wales Australia; ^6^ Department of Obstetrics and Gynecology McMaster University Hamilton Canada

## Abstract

**Objectives:**

Accurate diagnosis of pathology from ultrasound images is reliant upon images of a suitable diagnostic quality being acquired. This study aimed to create a novel machine learning model to automatically assess transvaginal ultrasound (TVUS) image quality for gynaecological ultrasound.

**Method:**

Six imaging professionals (two sonographers, two gynaecological sonologists and two radiologists) assigned a quality score to 150 TVUS images from 50 cases (50 uterus images and 100 ovary images). Images were given a score of 1–4 (1—reject/image inaccurate, 2—poor quality, 3—suboptimal quality or 4—optimal quality). As variation existed between the scores assigned by the labellers, we framed this problem as a multi‐annotator noisy label problem. To address this, a new machine learning architecture was developed, combining a weighted ensemble algorithm to estimate consensus labels and a multi‐axis vision transformer (MaxViT) to handle noisy labels, improving model accuracy in predicting image quality. Forty cases (120 images) were used for model training, while the remaining 10 cases (30 images) were reserved as a test set for model evaluation.

**Results:**

The novel machine learning architecture we created was able to successfully determine image quality with a validation accuracy of 80% and a macro average recall of 77%. This significantly improved upon the 57% accuracy of the baseline machine learning method (ResNet50). The MaxViTs were able to outperform human performance in most cases, with an accuracy of 80% surpassing four of the six human labellers.

**Conclusions:**

This novel machine learning model offers an automated method of assessing the quality of TVUS images. The tool has the potential to provide real‐time feedback to those performing TVUS, reduce the need for repeated imaging, and improve the diagnosis of gynaecological pathology.

**Summary**

- This study presents a novel machine learning model designed to automatically assess transvaginal ultrasound (TVUS) image quality, a critical factor in the accurate diagnosis of gynaecological pathology.
- By leveraging a weighted ensemble algorithm and a multi‐axis vision transformer (MaxViT), the model achieved 80% accuracy, surpassing traditional deep learning methods and outperforming human assessors in most cases.
- This innovation has the potential to provide real‐time feedback to sonographers, improve image acquisition standards, and reduce the need for repeat scans.
- Ultimately, this tool could enhance diagnostic accuracy, streamline workflows, and contribute to better clinical outcomes in gynaecological ultrasound.

## 1 Introduction

Artificial intelligence (AI) and machine learning (ML) are ever‐expanding tools within the healthcare sector, including gynaecology, that can aid in many tasks such as imaging interpretations, clinical decision making, and quality assurance (QA) [1]. For the investigation of gynaecological pathology, transvaginal ultrasound (TVUS) is the primary imaging tool. The quality of TVUS images significantly impacts diagnostic accuracy, influencing clinical decisions and patient outcomes, making image quality assessment (IQA) an essential part of a clinical TVUS examination. Image quality assessment is also an essential step in providing feedback to those learning to perform TVUS. In a typical clinical context, image quality (IQ) is often assessed (even if subconsciously) by the sonographer examining in real time to ensure diagnostically helpful imaging is obtained. This is then repeated by the reading clinician, who in the Australasian context of ultrasound is commonly a medical specialist who has not performed the real‐time examination. If the stored images (data) are not of sufficient quality, this may limit the accuracy of the interpretation or may result in a patient needing to be recalled for additional imaging. A worst‐case scenario is that a poor IQ could lead to an incorrect diagnosis and incorrect management for the patient.

Traditional methods of IQA often rely on evaluations by clinicians, which can vary [2]. Studies have shown that attempts to make human IQA more consistent, through mechanisms like structured checklists, are limited [3, 4, 5, 6]. The use of AI in IQA has not yet been documented within the application of TVUS for gynaecological imaging. However, given the potential of ML in medical imaging, it stands to reason that it could offers promise to address challenges with IQA, with several studies related to other aspects of medical imaging reported within the literature to date [7, 8, 9, 10]. Additionally, AI for IQA assessment has been utilised within medical photography–providing learnings which we can draw upon [11]. A study from Jeong et al. [11] for instance, used a VGG16 model to assess the quality of photos of skin lesions acquired on smartphones to determine whether they were suitable for diagnosis. While this novel approach has learning which we can apply to ultrasound, distinct differences exist, such as ultrasound being a modality of artefacts—many of which we use to assist with diagnoses and, therefore, do not wish to remove from images in many cases. Whereas a photo free of noise and, therefore, replicating what a clinician may examine with their own eyes during an examination is ideal. As such, approaches that are suitable for TVUS, or ultrasound more broadly, need a nuanced approach.

Despite the potential, developing robust ML models for the purpose of IQA is challenging due to the need for annotated datasets of a sufficient size for both training and validation. Given the variation in human‐led IQA, even very skilled experts performing labelling tasks may disagree on what the quality of any given image is. Such variations or inconsistencies in annotations from one or more labellers can introduce noise, creating significant challenges for the development of accurate models [12]. One possible avenue to overcome the challenge of noisy labelling is employing multiple annotators so that each image is given multiple labels to produce multi‐annotator classification. Given the differences in human interpretations, it is anticipated that these labels may differ. Common methods utilised to address labelling differences and reach a consensus label include majority voting or weighted voting [12, 13]. However, these approaches are often too simplistic to produce high‐quality estimates of the true label. This study aimed to create a novel ML model that can cope with noisy multi‐annotator labelling to automatically assess the quality of TVUS images.

## 2 Methods

### 2.1 Ethics Approval

Images used in the development of this algorithm were sourced from the IMAGENDO image library, for which ethical approval was granted by the University of Adelaide Human Research Ethics Committee (Approval number H‐2020‐051). Patients from whom the images were obtained provided their consent for images to be used in research involving the development of AI and ML tools. The study protocol was not published ahead of commencing the study. This study has been reported in keeping with the Transparent Reporting of a multivariable prediction model for Individual Prognosis or Diagnosis (TRIPOD‐AI) (Appendix S1) [14].

### 2.2 Dataset and Image Labelling

The dataset contained 150 TVUS images from 50 unique cases. Each case included an image of the left ovary, the right ovary, and the midsagittal uterus. Images were sourced from the stage one IMAGEDO data library, which contains studies from various clinical sites with a variety of ultrasound machines, performed by various operators. Images within this library date from 2012 to the present. Image labelling was performed by six ultrasound professionals: two sonographers, two radiologists, and two gynaecological sonologists. All labellers were very experienced in gynaecological ultrasound with experience ranging from 6 to 20 years. The mix of radiologists, sonologists, and sonographers provided a combination of those typically performing real‐time ultrasound examinations and those typically reviewing stored images and videos offline.

The labelling professionals utilised a predefined scoring system to allocate a score to each image, which they felt best represented the quality of the image. The allocated scores were 1 = reject (image inaccurate), 2 = image quality poor, 3 = image quality suboptimal, and 4 = image quality optimal (Appendix S2). All labellers reviewed and scored each image, producing a total of 900 labels (150 images each with six labels). Details of the scoring system and labelling process utilised for this dataset, and the associated interobserver agreement have been previously reported in another publication [2]. As scores 1 and 2 both reflected ‘non‐diagnostic’ images, prior to algorithm development, all images that were scored either 1 or 2 were merged into one class (score 1) to create just three scoring classes. This resulted in each image being scored either 1, 2 or 3, representing a poor, suboptimal or optimal quality image. As there was inconsistency between the scores allocated by each of our labellers, our dataset contained noisy labelling. The dataset size of 150 images was used for the development of this model due to the resource‐intensive nature of manual labelling. While the optimal dataset size for ML development varies according to the task and image complexity, this dataset size was considered a feasible starting point to explore model development and assess its potential utility.

### 2.3 Algorithm Building

**FIGURE 1:**
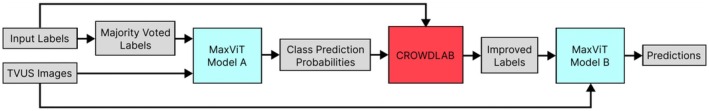
The architecture of the Multiaxis Vision Transformer (MaxVit) model utilised. MaxViT is a deep learning architecture that combines convolutional layers with transformer‐based self‐attention mechanisms. It incorporates both local and global feature representation, making it well suited to tasks requiring detailed spatial understanding such as ultrasound image interpretation.

Each image in the dataset was resized to a dimension of 224 × 224 pixels and normalised. All images were also augmented using AutoAugment [15] via the ImageNet augmentation policy. The proposed multiaxis vision transformer (MaxViT) model [16] utilised is pictured in Figure 1. The algorithm was developed with three distinct aspects: a relabelling model, a weighted ensemble algorithm, and a quality prediction model.

**FIGURE 2:**
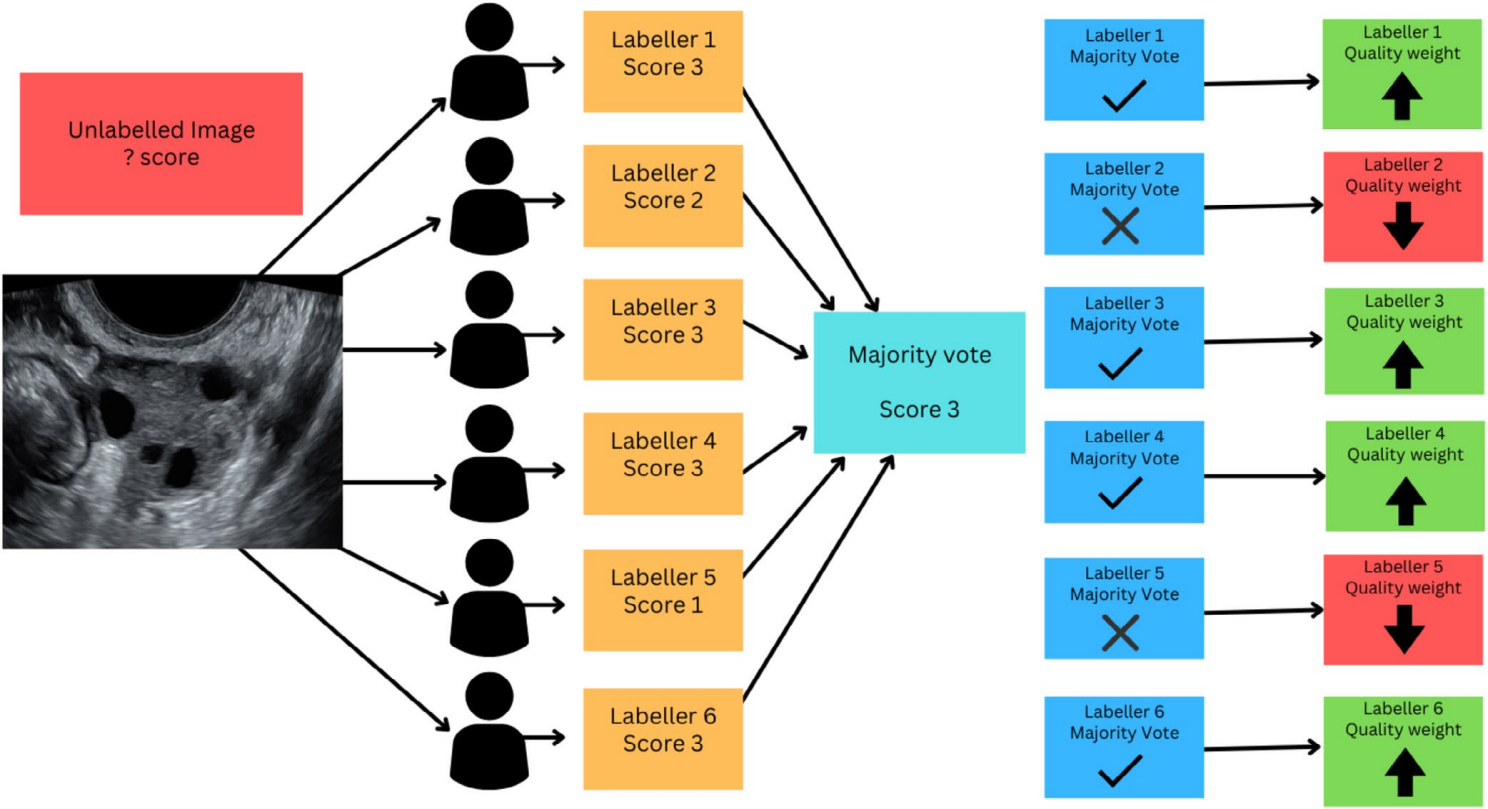
A high‐level graphic depiction of the process utilised to determine the labeller quality weighting. If the score assigned to an image matched the majority vote, the labeller's quality was determined to be good, and their quality weight went up. If the score assigned to an image did not match the majority vote, the labeller's quality was determined to be poorer, and their quality weight went down. This was repeated for each image in the dataset (*n* = 150) to determine the final labeller quality weighting.

The relabelling model was pre‐trained on ImageNet‐1K and then finetuned using our dataset of multi‐annotated TVUS images. Due to the limited size of the dataset, pretraining was a necessity. To manage the problem of noisy labelling, the first round of algorithm training was preceded by a majority vote to generate a consensus label for each image. We used CrowdLab's cleanlab.multiannotator models [13] that refines multi‐annotator data by generating consensus labels and annotator quality first, followed by a classifier to enhance label accuracy and assess annotator reliability. Annotator quality was assessed by comparing each annotator's label against the consensus label determined through majority voting. For instance, if the six professionals scored a particular image 2, 2, 3, 3, 2, 2; the image was allocated a score of two as most labellers allocated this score as the majority vote. Tiebreaks were resolved by randomly selecting one of the tied labels. Based on this score, we calculated annotator quality, meaning those who deviated from the majority vote were assigned a lower weighting. A high‐level description of this process is provided in Figure 2.

**FIGURE 3:**
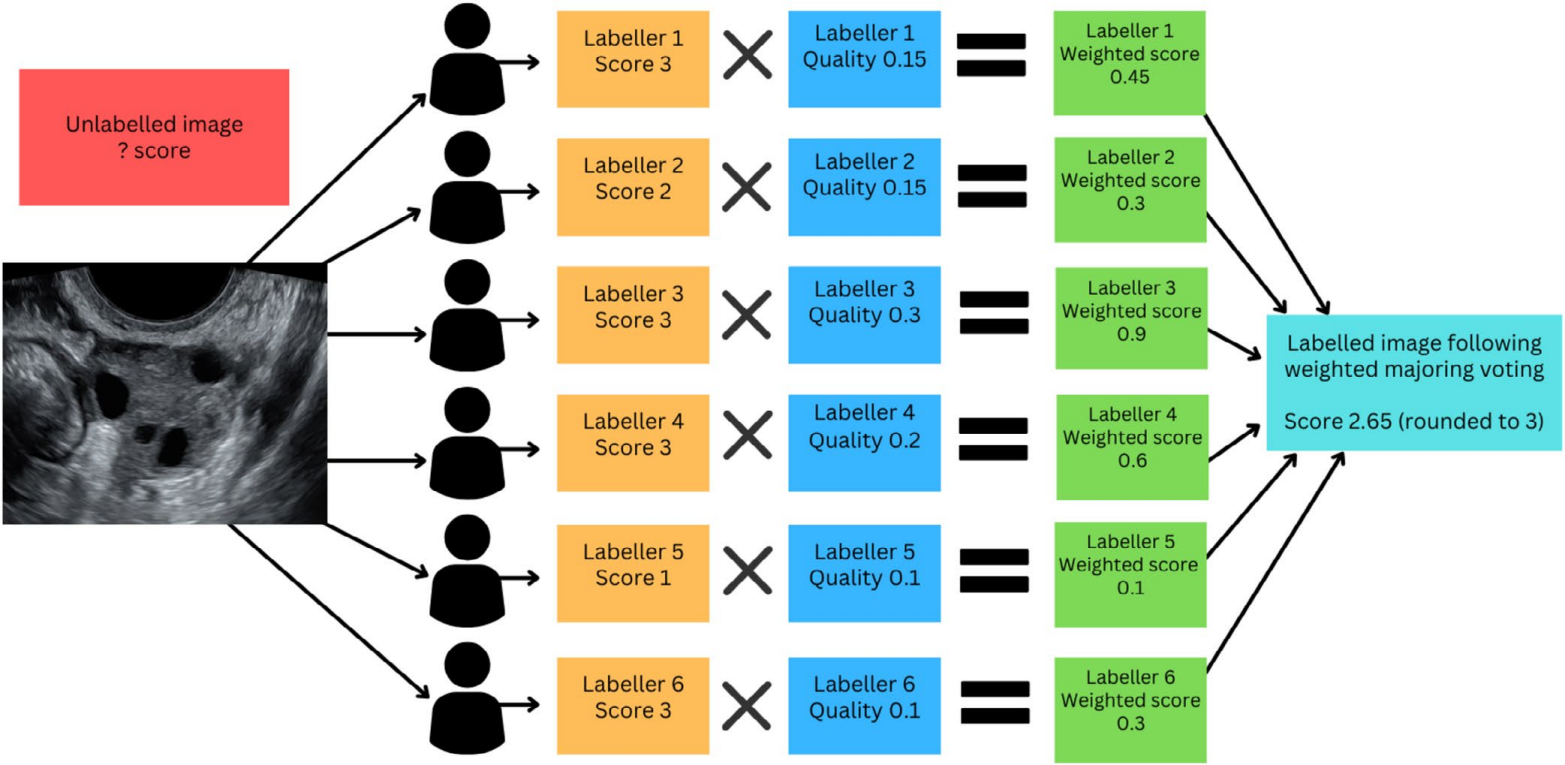
A high‐level graphic depiction of the process utilised to create a weighted majority vote label for each image based on the scores assigned by each of the six labellers. The score assigned by the labeller was multiplied by the labeller's quality score to create the new weighted score. Following this, the six weighted scores were added to create the weighted majority vote score.

Following this, CrowdLab performed weighted majority voting to determine the final label, which was then used to train the ML model (Figure 3). The weighted ensemble algorithm utilised the class prediction probabilities from the relabelling model and the multiple annotators' labels to improve the consensus label of each sample. Finally, the quality prediction model was pretrained on ImageNet‐1K [17] and then finetuned using the improved consensus labels. The detailed coding framework underpinning the algorithm development is described in greater detail in a prior conference presentation [18].

Forty cases (120 images) were used as the training set, with the remaining 10 cases (30 images) reserved as the validation set. These cases were selected to ensure the distribution of image quality was consistently the same across the training and validation sets. The dataset was unbalanced, with 351 score 3 labels, 331 score 2 labels, and 218 score 1 label, with score 1 being underrepresented, making up only 24.2% of annotations. This imbalance was exacerbated when observing the majority‐voted labels, which were 27 (18.0%) score 1 labels, 52 (34.7%) score 2 labels, and 71 (47.3%) score 3 labels.

### 2.4 Training/Validation Process

The MaxViT was trained on our TVUS dataset for 100 rounds of learning. During training, it observed groups of four examples at a time, adjusting its settings to better understand the data, using a method called AdamW optimisation [19]. It also used prior knowledge from a pre‐existing dataset (ImageNet‐1k) [17] to accelerate learning. ImageNet‐1k is a widely used benchmark for visual recognition. While it is known to contain some labelling errors and inherent biases, its application in our study was limited to providing initial pre‐trained weights for our model. The model was subsequently fine‐tuned and validated using our own high‐quality, expert labelled scans. The fine‐tuning process minimises the influence of the potential bias in the ImageNet pre‐training weights by adapting the model to recognise the specific features and statistical distributions of our target domain.

The trained MaxViT model was then combined with an additional technique called weighted ensemble to further improve its performance. Following this, the algorithm was fine‐tuned for another 100 rounds. We evaluated the algorithm using 5‐Fold Cross‐Validation to reduce the risk of overfitting to a specific validation set and provide a more accurate estimate of model generalisation.

### 2.5 Performance Analysis

Performance of our proposed model was compared with majority voting on the TVUS dataset using Resnet50, [20] Resnet101 [20] and MaxViT. The accuracy and macro average recall (sensitivity) were calculated for each architecture as well as per class precision, recall, and F1‐score (the harmonic mean of precision and recall). ResNet‐50 and ResNet‐101 are deep convolutional neural networks (CNNs), with 50 and 101 layers respectively, that utilise residual learning to enable deep architectures to train effectively. Residual connections help prevent vanishing gradients, allowing the network to learn more complex features. These models are widely used in image classification tasks and serve as strong baselines in medical imaging [20].

## 3 Results

The overall ML model performance can be seen in Table 1. The validation accuracy of the proposed algorithm was 80% and the macro average recall was 77%. Resnet50 had an accuracy of 63% and macro average recall of 65%. Resnet101 had a similar accuracy of 60% and macro average recall of 60%. Each model's accuracy and macro average recall improved when changing from majority vote to our proposed algorithm. F1 scores were calculated to further assess performance.

**TABLE 1:** Performance of our proposed algorithm with majority voting in the transvaginal ultrasound quality scoring dataset using Resnet50, Resnet101 [20] and MaxViT.

Model	Consensus label	Accuracy	MacroAverage R	Quality score 1			Quality score 2			Quality score 3		
				Precision	Recall	F1‐score	Precision	Recall	F1‐score	Precision	Recall	F1‐score
Resnet50	MV	0.57	0.59	0.67	0.86	0.75	0.38	0.33	0.35	0.62	0.57	0.59
	WE	0.63	0.65	0.50	0.75	0.60	0.67	0.50	0.57	0.67	0.71	0.69
Resnet101	MV	0.53	0.53	0.56	0.71	0.63	0.33	0.22	0.27	0.60	0.64	0.62
	WE	0.60	0.69	0.50	1.00	0.67	0.64	0.58	0.61	0.64	0.50	0.56
MaxViT	MV	0.63	0.64	1.00	0.71	0.83	0.45	0.56	0.50	0.64	0.64	0.64
	WE	0.80	0.77	0.75	0.75	0.75	0.88	0.64	0.74	0.78	0.93	0.85

Abbreviations: MV, majority voting; WE, weighted ensemble.

Despite the imbalanced dataset, each model's accuracy and macro average recall were similar, indicating that performance was not biased towards any one of the three scores. The F1 scores of MaxViT with weighted ensemble were 75%, 74%, and 85% for score 1, score 2, and score 3 respectively, indicating balanced performance across all scores. MaxViT had the most significant improvement in both accuracy and macro average recall when using weighted ensemble compared to majority voting. The labels produced by the weighted ensemble algorithm (across MaxViT, Resnet50, and Resnet101) led to a significantly increased F1 score for score 2 (an increase of 50%–74%) and score 3 (an increase of 64%–85%), score 1 F1 score decreased from 83% to 75%.

The MaxViT's performance was further evaluated against the human‐labellers' performance using the weighted ensemble algorithm's labels (Table 2). The MaxViT was able to outperform human performance in most cases, with an accuracy of 80% surpassing four of the six human labellers.

**TABLE 2:** Performance of the MaxViT compared to the human labellers using the weighed ensemble algorithm's labels.

Accuracy	
Model performance	
MaxViT	0.80
Human performance	
Labeller 1	0.97
Labeller 2	0.90
Labeller 3	0.73
Labeller 4	0.70
Labeller 5	0.60
Labeller 6	0.40

## 4 Discussion

This paper presents a novel ML model for automated IQA of TVUS images, which utilised a dataset labelled by multiple annotators, who all showed variation in their interpretation. This variation led to ‘noisy labelling’ and therefore, our model was developed to specifically navigate this complication of image labelling for IQA. To the best of our knowledge, this represents the first report within literature of an ML model assessing the image quality of TVUS images specifically. Our proposed model was able to surpass the performance of humans in most cases suggesting that ML (AI) could be a very valuable tool for IQA moving forward.

Quality assessment of ultrasound images by human operators is variable, which is a well‐documented challenge within the literature [3, 4, 6, 21, 22, 23, 24]. Inconsistency not only exists between different human operators, but intraoperator variation in quality scoring has been reported when the same operators reviewed the same images on separate occasions [2]. This intraoperator variation may be explained by factors affecting human performance and concentration, such as distractibility and fatigue [1]. As AI systems are not affected by such human factors, they may offer advantages over the human assessment of IQ and, if well trained, hold potential to perform more consistently than humans, as our results show.

Despite the prospects of AI systems for IQA, the development of such models is challenging for several reasons. As with any AI model, large, labelled data sets are needed [1]. When aiming to build AI tools to assist diagnosis, this is often binary (i.e., disease is present, or disease is not present) and AI tools are well‐suited to this type of classification. Quality assessment, however, is a *shade of grey* and, as such, quality labelling of data by humans will have inconsistencies [22]. The result of this will be noisy image labelling, which is typically problematic for AI tools to manage [12, 22]. Strategies like transfer learning (using models pre‐trained on large datasets and fine tuning for the smaller, available datasets), open‐source data sharing and further development of standardised image acquisition and annotation protocols at a professional level will all likely have a role to play in helping to overcome these limitations as the role of AI in ultrasound continues to expand. In addition, innovative data labelling strategies will also help to mitigate against these challenges [25]. Our proposed model specifically aimed to utilise ‘noisy labels’ with a weighted ensemble method to improve upon consensus labelling. Other potential solutions to ‘noisy labelling’ challenges include active learning or semi‐supervised learning approaches, where experts provide feedback only on the most challenging cases, helping to refine AI performance [26, 27]. Although this is the first study within the literature proposing an ML IQA tool specific to TVUS, similar systems have been developed pertaining to other areas of ultrasound imaging which have attempted to address the challenge of noisy data. Raina et al. [28] proposed an UnSupervised Ultrasound image Quality assessment Network (US2QNe) to automate IQA assessment of images of the urinary bladder. Their model achieved a similar accuracy to ours (78%). Similarly, Liao et al. [22] proposed a novel solution to the aleatoric uncertainty inherent in ultrasound image labelling (which they referred to as *an aleatoric uncertainty modelling regression problem*). Although their dataset was echocardiography images, their simple model increased accuracy by 5.7% and could be generalised to other ultrasound applications like gynaecological ultrasound.

Automated IQA systems hold great potential to be a clinically beneficial application of AI in gynaecology ultrasound on various levels. Broadly, a simple to apply system of checking image quality at the time of the examination holds potential to reduce the need to recall patients (saving time and cost to patients and clinics alike) and increase diagnostic confidence and accuracy for those interpreting imaging. Other applications may include departmental or governing body level QA programmes. Quality auditing is a well‐established method of maintaining high standards and safeguarding against potential errors [29]. Human‐led audits for QA purposes are logistically difficult and time‐consuming, which limits how frequently they can be performed [29]. Automating audits removes these challenges and opens the possibility for auditing to occur far more frequently without the need to allocate extensive resources.

Automated IQA systems in gynaecological ultrasound hold potential to be used for teaching and training purposes, with automated IQA systems able to assess IQ and report this back to sonographers in real‐time. Such a system has existed commercially for several years in fetal ultrasound (ScanNav, Intelligent Ultrasound, Cardiff, UK) [30] which is now available as a Food and Drug Authority (FDA) and Conformite Europeenne (CE) approved package (SonoLyst) within GE ultrasound systems (GE Healthcare, Chicago, United States of America). While the SonoLyst system uses IQA to enhance the accuracy of segmentation, sophisticated multitasking systems also hold great potential for medical ultrasound whereby automated IQA can act as a thresholding tool for AI tools providing image interpretation for assistance in diagnostic clinical decision making [31] For instance, the initial step of using an AI diagnostic aid could involve having the image ‘read’ by an IQA tool to determine whether the quality is sufficient for diagnosis, maintaining high levels of diagnostic accuracy. Such systems exist in the field of echocardiography, encompassing fully integrated multitasking systems to perform IQA [22, 32, 33] segmentation and image interpretation [34, 35] This work has led to autonomous systems which are now licensed by the FDA and available for use clinically [36] suggesting that this too could be a future reality in gynaecology.

A limitation of the study was the limited size of our dataset which may have failed to capture the full spectrum of variability present within clinical settings. Furthermore, as our model was evaluated on only 30 images, drawing statistically significant conclusions from this single study is not possible. However, as the intention of this study was to produce a ‘proof of concept’, the sample size was sufficient to meet this aim. Future studies to further refine this model would be helpful and external validation of our model with other datasets is required to assess its suitability for clinical use.

While our data was unbalanced, due to the overrepresentation of score 3 (optimal quality images), a strength of this study was the use of a range of ultrasound images from various operators and different makes of ultrasound machines.

## 5 Conclusion

In this paper, we introduced a novel ML model that harbours potential to automate the quality assessment of TVUS images. The distinctive architecture of our ML model effectively utilised a pre‐trained MaxViT in conjunction with annotations from multiple labellers. This approach allowed the model to cope with variation in labelling between operators, yielding a prediction accuracy of 80%, which was more consistent than humans. With further refinement of this model through additional training with larger datasets, accuracy may be improved. The model we created holds potential to be turned into a clinically beneficial tool in the future, assisting with tasks such as providing feedback to those learning to perform TVUS or performing QA assessments. This model could also be used in the creation of multitasking AI tools for clinical decision making and imaging interpretation. This work contributes to the rapidly evolving field of AI‐assisted ultrasound technology and offers a potential solution to the natural variability in IQA that arises from human interpretation.

## Author Contributions

Conceptualisation: Alison Deslandes, Daniel Petashvili, Hu Wang, Gustavo Carnerio, Jodie Avery, George Condous, Mathew Leonardi, M. Louise Hull, Hsiang‐Ting Chen. Data curation: Alison Deslandes, Daniel Petashvili, Jodie Avery. Formal analysis: Alison Deslandes, Daniel Petashvili, Hu Wang, Gustavo Carnerio, Hsiang‐Ting Chen. Investigation and Methodology: Alison Deslandes, Daniel Petashvili, Hsiang‐Ting Chen. Project administration: M. Louise Hull, Hsiang‐Ting Chen, Gustavo Carnerio, Jodie Avery. Resources: Jodie Avery, M. Louise Hull, Hsiang‐Ting Chen. Software: Daniel Petashvili, Hu Wang, Gustavo Carnerio, Hsiang‐Ting Chen. Supervision: M. Louise Hull, Hsiang‐Ting Chen, Gustavo Carnerio, Jodie Avery, George Condous. Validation: Alison Deslandes, Daniel Petashvili, Hu Wang, Gustavo Carnerio. Visualisation: Daniel Petashvili, Alison Deslandes. Writing – original draft: Alison Deslandes, Daniel Petashvili. Writing – review and editing: All authors.

## Disclosure

*Presentation of Work at Conferences*: Elements of this work were presented at the 2024 IEEE International Symposium on Biomedical Imaging (ISBI) in Greece and the 2024 ISUOG World Congress in Budapest, Hungary. These abstracts/papers can be found online at these links: https://ieeexplore.ieee.org/abstract/document/10635761 and https://obgyn.onlinelibrary.wiley.com/doi/full/10.1002/uog.29025.

*Preprints*: This work has not been published as a pre‐print on any platform.

## Conflicts of Interest

This work has been performed as part of the IMAGENDO project. The University of Adelaide holds a provisional patent related to the broader work of the IMAGENDO group in this field, with authors Prof M. Louise Hull, Prof George Condous, Dr. Mathew Leonardi, Dr. Hu Wang and Prof Gustavo Carneiro listed as inventors on the patent. However, the content and findings presented in this manuscript do not pertain directly to the subject matter of the provisional patent. The authors confirm there are no conflicts of interest that could have influenced the design, execution, or interpretation of the research described in this article.

## Supporting information


**Appendix S1:** ajum70026‐sup‐0001‐AppendixS1.pdf.

**Appendix S2:** ajum70026‐sup‐0002‐AppendixS2.docx.

## Data Availability

The raw data used in the development of this algorithm are not publicly available due to ethical requirements. The analytical code used can be made available upon reasonable request to the authors.
