# Supplementary material for: Development of a Novel Machine Learning Model for Automatic Assessment of Quality of Transvaginal Ultrasound Images From Multi‐Annotator Labels
Source: Australas J Ultrasound Med. 2025 Sep 22;28(4):e70026. doi: 10.1002/ajum.70026 (PMC12454678; doi:10.1002/ajum.70026)
Supplement: Supplementary file 1 [file AJUM-28-0-s001.pdf]

Appendix S1

Scoring system used for image labelling

Table 1: The scoring system utilised for image labelling. Images were assessed against the five factors listed and assigned a score of 1-4 for each factor. A score of 4 represents optimal image quality while a score of 2 represent poor image quality.

1 (Reject)	2	3	4
**Image inaccurate**	**Image quality poor**	**Image quality suboptimal**	**Image quality optimal**
	Correct anatomy not confidently recognisable	*Correct anatomy recognisable*	*Correct anatomy easily recognisable*
	Some of the anatomical structure is not seen	*Most of the anatomical structure is clearly seen*	*Entire anatomical structure clearly seen*
	Unacceptable image optimisation (depth, focus, gain)	*Acceptable image optimisation (depth, focus, gain)*	*Good image optimisation (depth, focus, gain)*
	Unable to interpret image for diagnosis	*Possible to interpret image for diagnosis*	*Easy to interpret image for diagnosis*
	Overall image clarity is poor	*Overall image clarity is satisfactory*	*Overall image clarity is good*

Explanatory notes

Images were assigned a score of 1 (quality poor), 2 (quality suboptimal) or 3 (quality optimal) based on the five criteria outline in Table 1. If an image was inaccurate (e.g. the image should have demonstrated an ovary but demonstrated the cervix, etc), it was assigned a score of 1. The criteria by which the grades were assigned are;

a) Ability of anatomy to be recognised in image,
b) The clarity of the anatomical structure in field of view,
c) Image optimisation (depth, focus and gain),
d) Ability of image to be interpreted for diagnosis of pathology, and
e) The overall clarity of the image.
